# Effect of Some Biopolymers on the Rheological Behavior of Surimi Gel 

**DOI:** 10.3390/molecules17055733

**Published:** 2012-05-14

**Authors:** Md. Zaidul Islam Sarker, M. Abd Elgadir, Sahena Ferdosh, Md. Jahurul Haque Akanda, Mohd Yazid Abdul Manap, Takahiro Noda

**Affiliations:** 1 Department of Pharmaceutical Technology, Kulliyyah of Pharmacy, International Islamic University, Kuantan Campus, Kuantan, Pahang 25200, Malaysia; Email: zaidul@iium.edu.my; 2 Department of Pharmaceutical Pharmacology and Chemistry, Faculty of Pharmacy, Universiti Teknologi Mara, Shah Alam, Selangor 42300, Malaysia; Email: saeedfood93@yahoo.com; 3 School of Industrial Technology, Universiti Sains Malaysia, Minden, Penang 11800, Malaysia; Email: sahenaferdosh@gmail.com (S.F.); jahurulhaque@yahoo.com (M.J.H.A.); 4 Department of Food Technology, Faculty of Food Science and Technology, Universiti Putra Malaysia, UPM Serdang, Selangor 43400, Malaysia; Email: myazid@food.upm.edu.my; 5 Hokkaido Agricultural Research Center, National Agriculture and Food Research Organization, Shinsei, Memuro, Kasai, Hokkaido 082-0081, Japan

**Keywords:** rheological properties, surimi, starch, cryoprotectant, mannan

## Abstract

The objective of this study was to investigate the effect of selected biopolymers on the rheological properties of surimi. In our paper, we highlight the functional properties and rheological aspects of some starch mixtures used in surimi. However, the influence of some other ingredients, such as cryoprotectants, mannans, and hydroxylpropylmethylcellulose (HPMC), on the rheological properties of surimi is also described. The outcome reveals that storage modulus increased with the addition of higher levels of starch. Moreover, the increasing starch level increased the breaking force, deformation, and gel strength of surimi as a result of the absorption of water by starch granules in the mixture to make the surimi more rigid. On the other hand, the addition of cryoprotectants, mannans, and HPMC improved the rheological properties of surimi. The data obtained in this paper could be beneficial particularly to the scientists who deal with food processing field.

## 1. Introduction

According to Park and Morrissey [1], surimi is a concentrate of myofibrillar proteins obtained after mincing and washing fish flesh. Lanier [2] reported that, from a physical and chemical viewpoint, surimi is light in color with a bland odor, low in fat, and has high myofibrillar protein. Due to the existence of myofibrillar protein, surimi has unique gelling properties that make it an excellent functional ingredient for manufacturing food products. Traditionally, surimi is prepared from the muscle of some specific species, such as Pacific whiting (*Merluccius productus*) and Alaska pollock (*Theragra chalcograma*). In addition, various other species such as bigeye snapper (*Priacanthus* spp.), treadfin bream (*Nemipterus* spp.), lizardfish (*Saurida* spp.), croaker (*Pennahia* and *Johnius* spp.), are now also used in the preparation of commercial surimi in southeast Asia; the functional and compositional properties vary depending on the species used [3].

Surimi is a unique and useful seafood analogue because of its gelling properties [3,4]. Since viscoelasticity is an important quality of surimi products, the ingredients used in their preparation are significant factors influencing the rheological properties of surimi. Starch has been reported as the most important ingredient in surimi seafood production [5,6,7,8,9,10]. According to Yang and Park [7], thermal processing causes many changes in starch properties, such as granule swelling, more water absorption, and imparting of viscosity. Starches vary in texture in surimi-starch mixtures due to their botanic sources. For instance, surimi gels containing potato starch were firmest and most cohesive [6]. Several authors have reported the influence of mixing of other ingredients, such as cryoprotectants [11], mannans [12], hydroxylpropylmethylcellulose (HPMC) [13] on the rheological properties of surimi products. Several cryoprotectants, namely sugars, are added to surimi products to enhance certain functional properties, such as protection from freezing damage. For instance, low-molecular-weight sugars, such as trehalose [14], sorbitol [15], sucrose [16], and glucose [17], are well documented as the most effective in cryostabilizing isolated proteins and surimi. Sugars are also used to investigate the thermal properties of frozen and freeze-dried surimi products, such as the glass transition [18]. In this paper, we describe the influence of selected biopolymers on the rheological properties of surimi products.

## 2. Surimi-Starch Mixture

**Figure 1:**
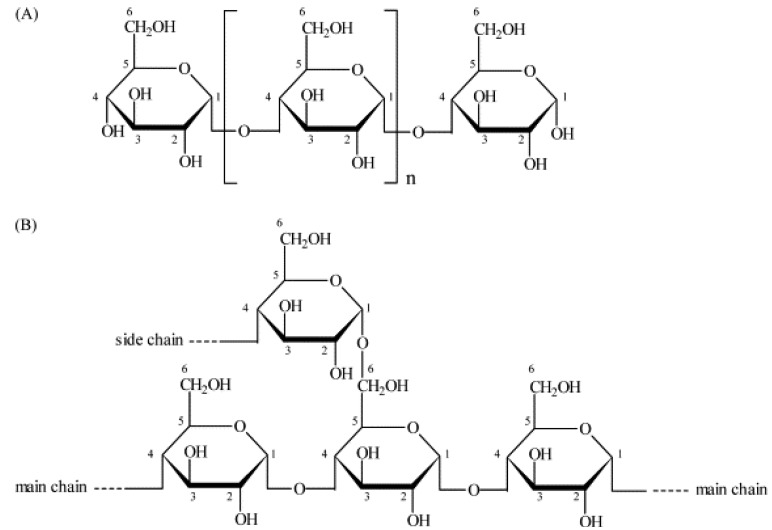
Structure of starch amylose (**A**) and amylopectin (**B**) (adapted from Herrero-Martínez *et al.* [28]).

Starch is made up of two polysaccharides, amylose and amylopectin (Figure 1). Amylose is a linear or slightly branched polymer consisting of α-1,4-linked glucan chains with a few branch [19,20]. Amylopectin has large molecular weight and highly branched structure constructed from hundreds of short α-1,4 linked glucan chains, which are interlinked by α-1,6 linkages [21,22]. Starch normally contains 15–30% amylose and 70–85% amylopectin. However, waxy starch contains no or very little amylose, and a few starches from genetic variants of the pea [23] and maize [24,25] definitely contain high amylose contents (>50%). Starch granules are considered to have alternative layers of crystalline and amorphous regions. The crystalline regions are believed to be densely packed structures, which are derived from the regions of amylopectin branches. Amylose appears to be present mainly in the amorphous regions of starch granules [26]. As an aqueous starch is gradually heated in the presence of sufficient water, irreversible swelling of starch granules occurs. It was reported that the starch granules absorbed more water and the starch viscosity increased dramatically with an increase in the heating temperature before reaching the gelatinization temperature [27]. 

The chemical, physical, and functional properties of starches have been widely studied. Native starches from various botanical sources, such as maize, sweet potato, potato, and rice, have received extensive attention due to the differences in their structural and physico-chemical characteristics [29,30,31]. In food production systems, starches are widely used, and they contribute greatly to the textural properties of the food as gelling agents, thickeners, water retention agents, textural agents, and colloidal-stabilizer bulking agents. Thus, it is critical to analyze the functional properties of starch, such as pasting and gelatinization properties, for the high utilization of starch-based food products. The ratios of amylose to amylopectin determine several functional characteristics, such as pasting and gelatinization properties and swelling power [24,25,31,32,33,34]. The molecular structure of amylopectin also affects physico-chemical properties of starch [30,31,35,36,37,38,39]. The functional properties of starch in food mixtures are determined by their interactions with other food components, their transformations upon hydrothermal processing, and aging [40,41]. Partial solubilization of starch granules due to the swelling upon heating in water contributed mostly to the structure of starch. Moreover, amylose and amylopectin have a poor miscibility and unequal affinity towards water, causing starch to have separate phases. The presence of other ingredients, such as polysaccharides and proteins, as polymeric materials lead to the formation of a third phase besides the other two starch phases. This behavior could be due to the limited thermodynamic miscibility of the different pairs of polymers [42,43].

Normally, consumers evaluate the final acceptance of surimi-based products by their textural characteristics, which are considered to be most important. When a surimi-based product contains high-quality surimi as a predominant component, the resulting texture of the product tends to be rubbery [44]. Lee *et al.* [44] also claimed that ingredients must be added to surimi in order to modify its water mobility and textural properties. This is done to make the surimi product more desirable to consumers. As a functional food additive, starch is valued because of its contribution to texture. Various starches have been included in meat products, such as surimi, for many purposes, such as improving the textural characteristics and reducing the cost of the products [8]. Lee *et al.* [44] noted that the addition of starch into surimi seafood products resulted in the modification of product texture, reductions in cost, and improvements in freeze-thaw stability. Starch has been recognized as a filler in surimi, and it is used to increase firmness of products, as reported by Verrez-Bagnis *et al*. [45]. The level and type of starch affect both the chemical and physical properties and the structure of surimi gel. Lee *et al.* [44] added various amounts of potato starch to surimi sol gel and investigated the effects on the rheological properties of surimi before and after gelation. They found that the tensile and compression properties were a function of the level of starch added [44]. The addition of starch caused an increase in gel strength, which was attributed to the formation of elastic starch globules increasing the density of the protein matrix. Park [5] reported that, when surimi was partially replaced by starch, a significant increase in product gel strength was observed. The addition of pre-gelatinized starch to surimi products has caused decreases in both the water-holding capacity and firmness compared to control (no addition of starch) as well as that of intact starch [46]. The botanical origin of starch also affects the rheological properties of surimi. For example, among the starches from different botanical origin, potato starch with a high viscosity and good water-holding ability has a greater strengthening ability of starch/surimi system [6]. Yang and Park [7] studied the effects of starch modification on the texture of surimi-starch gels and found increasing gel strength of surimi-starch mixtures for starches modified with acetylation. Tuankriangkrai and Benjakul [47] reported the effect of modified tapioca starch (hydroxylpropylated distarch phosphate) on the textural and sensory properties of bigeye snapper mince gels subjected to different freeze-thaw cycles. It was concluded that the addition of 1% of modified starch could maintain the quality of gel products during extended frozen storage.

During thermal processing of starch-surimi systems, significant changes in rheological properties were observed due to the gelatinization of starch and sol-gel transformation of fish proteins, using various starches [48,49]. Wu *et al.* [48,49] reported that the starch granules present in surimi-starch and starch-water systems behave differently when thermally treated. This thermal behavior could be definitely delayed by the presence of some ingredients, such as sucrose, salt and myofibrillar proteins, in a surimi-starch system. The denaturation of myofibrillar proteins during thermal treatment occurred before the completion of starch gelatinization. The availability of water for starch gelatinization was limited because the water was entrapped in the protein-gel network and, as a result, competition for water between starch and proteins occurred. Thus, the gelatinization of starch was an important factor in the formation of the network structure of surimi-starch gels. 

In the study of Chen and Huang [13], surimi with a high water content (84% and 86%) was mixed with potato starch with the purpose of examining the gelation properties of the system. The authors observed a higher storage modulus (*G*′) value for surimi with a higher addition of starch [13]. They also found, during thermal scanning for the starch-enhanced surimi, that the value of *G*′ did not decrease during the gel resolution stage [13]. The addition of starch resulted in increasing gel strength, breaking force, and deformation of surimi. A minimum concentration of 10% starch was added to surimi with an 86% water content to detect the gel strength in the mixture. Starch granules act as a filler reinforcement and water-absorbent agent in surimi. The reason for the increase in the *G*′ value was the absorption of water by starch granules in the surimi-starch mixture, resulting in more rigid surimi. Campo-Deaño and Tovar [10] investigated changes in the viscoelastic properties (*G*′ and loss modulus (*G*″)) of sticks prepared from Alaska pollock (AP) and Pacific whiting (PW) surimi mixed with starch at concentrations of 7%, 11%, and 15%. The difference of (*G*′–*G*″) had two distinct regions from a rheological viewpoint, namely, a linear viscoelastic region in which the *G*′ value was greater than the value of *G*″ and a non-linear region in which the value of *G*′ decreased with increasing stress or strain and the *G*″ value exhibited a contrasting trend [10]. These results, according to Bruno and Moresi [50] and Moresi *et al.* [51], could be explained by the limits of the linear viscoelastic range in terms of shear stress (*σ*_max_) or strain (*γ*_max_). Campo-Deaño and Tovar [10] found that in general, as the starch concentration increased, the viscoelastic moduli and gel strength, expressed as the complex modulus (G*), also increased. The authors also observed that, with identical starch concentrations, the PW samples had lower viscoelastic moduli than the AP samples [10]. This result proves that increased resistance in shear forces from AP gels was more rigid than that in those from PW.

## 3. Surimi-Cryoprotectant Mixtures

A cryoprotectant is usually used to protect against damage of biological tissue from ice formation. Some cryoprotectants function by lowering the glass transition temperature of the solution or material. Many cryoprotectants function by forming hydrogen bonds with biological molecules like water molecules in a system. However, cryprotectants prevent freezing, and, in a glassy phase, a solution can maintain some flexibility. For proper DNA and protein function, hydrogen bonding in aqueous solutions is important. Thus, the biological material retains its native physiological structure, although it is no longer immersed in an aqueous environment because the cryoprotectant replaces the water molecules. During frozen storage, surimi is liable to lose quality due to denaturation and/or aggregation of myofibrillar proteins. Cryoprotectants prevent the protein, especially actomyosin, from denaturing/or aggregating. Thus the addition of cryoprotectant to surimi is needed to maintain its quality. Many compounds, including some low molecular weight sugars and polyols as well as many amino acids, carboxylic acids and polyphosphates were known to display cryoprotective effects in surimi [11,14,15,16,17,52,53,54,17,52]. Sucrose and sorbitol are the cryoprotectants commonly used in the surimi industry. Campo-Deaño *et al.* [11] compared the effect of adding different cryoprotectants (sorbitol + sucrose, sorbitol + trehalose and trehalose alone) on surimi prepared from the muscle of giant squid (*Dosidicus gigas*). Two methods were used to prepare surimi: first, protein precipitation at the isoelectric point (type A), and, second, washing with an acid solution (type B). Four % sorbitol + 4% sucrose + 0.5% sodium tripolyphosphate, 4% sorbitol + 4% trehalose + 0.5% sodium tripolyphosphate, and 8% trehalose + 0.5% sodium tripolyphosphate were added to the surimi of type A or B. Viscoelastic parameter studies showed that surimi type A samples had significantly higher viscoelastic moduli than type B samples and were more rigid than type B samples. In type B samples, the influence of different cryoprotectants was not discernible. In contrast, in type A samples, trehalose favored less initial protein aggregation and therefore a more thermorheologically stable structure.

The rheological properties of squid surimi (*Dosidicusgigas*) made by two methods (isoelectric precipitation, type A, and acid washing, type B) and stored frozen at −15 °C for 6 months were investigated by Campo-Deaño *et al.* [54]. Rheological tests were conducted using dynamic oscillation at constant temperature (10 °C) and temperature sweep tests from 10 to 90 °C. They reported that gel weakening, *G*′ and *G*′′ moduli, decreased between 45 and 50°C, reflecting an increase in fluidity of the semi-gel [54]. Some already formed protein networks may be disrupted, and the gel elasticity decreases. On the other hand, denaturation of actin could be ascribed to an increase in the number of both cross-links between protein aggregates and strands due to gel strengthening from 50 to 65 °C. Campo-Deaño *et al.* [54] also reported that, up to *T* > 70 C, *G*′ continues increasing, whereas *G*′′ remains almost constant, indicating the formation of a highly elastic myofibrillar protein gel. Moreover, in type A samples, there were some differences among the different cryoprotectants: in the type A sample containing sorbitol + trehalose caused a significant increase in *G*′ and *G*′′ moduli after eight weeks of storage of type A surimi throughout the temperature range. Sorbitol + trehalose had a weak cryopreservative effect on surimi *A*; in this case, the cryoprotectant may have increased the internal mechanical stress produced by freezing-induced dehydration. 

## 4. Surimi-Mannans Mixtures

**Figure 2:**
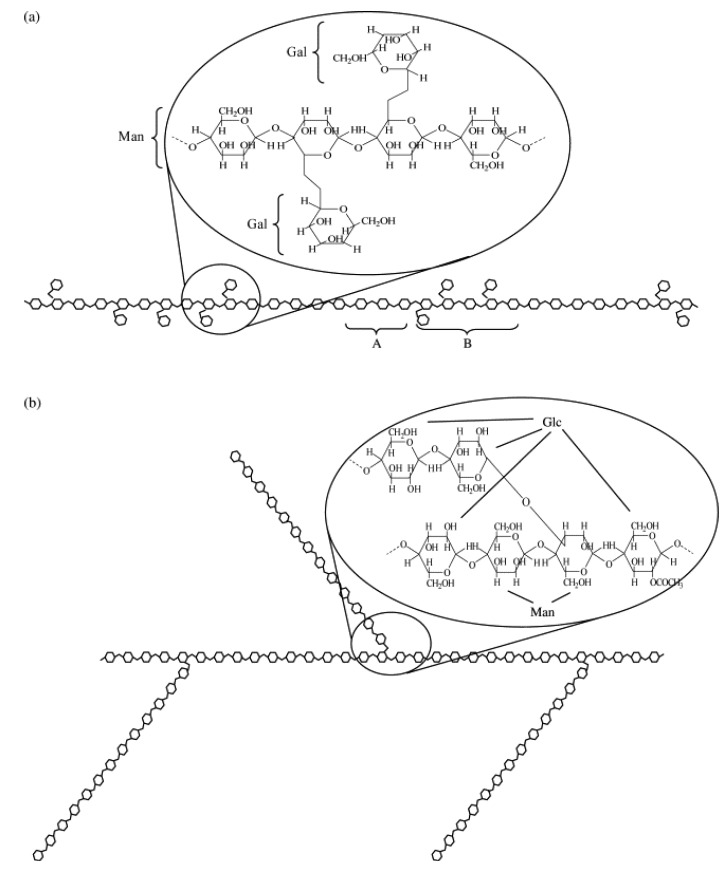
General structure of galactomannan (**a**) and glucomannan (**b**) (adapted from Vieira and Gil [57]).

Galactomannans and glucomannans are polysaccharides of the mannan family widely used in the food industry as thickeners and gelling agents. Galactomannans are composed of a β-1,4-linked d-mannan backbone with α-1,6-linked d-galactose side groups making up an approximate mannose/galactose ratio of 3.0 [55], whereas glucomannans are composed of β-1,4 linked d-mannose and d-glucose (mannose/glucose ratio = 1.6) with branches 11–16 residues long, occurring every 10–11 residues in the main chain [56] (Figure 2). For example, two types of galactomannans are commonly used in ice cream to improve texture and reduce ice cream meltdown, i.e., guar gum (GG) and locust bean gum (LBG). Dickinson [58] reported that galactomannan is assumed to function by modifying the rheological properties in the aqueous phase between the dispersed particles and droplets. The number of water-soluble polysaccharides with different chemical structures provides a range of functional properties and accounts for their extensive use in the food industry, such as creating a gel structures, enhancing viscosity, forming films, reducing ice crystal growth, inhibiting syneresis, lengthening physical stability, and improving textural properties [58]. Konjac glucomannan (KGM) is the main component of Konjac flour. Natural KGM is partially acetylated and forms a thermally irreversible gel by heating in the presence of an alkaline coagulant [59]. KGM has been introduced in the USA and Europe as a food additive. 

The effect of KGM on myofibrillar protein from grass carp (*Ctenopharyngodon idella*) during frozen storage at −18 °C and the influence of five levels of KGM (0%, 0.5%, 1%, 1.5%, and 2% w/w) on the textural properties of grass carp surimi gels were investigated by Xiong *et al.* [12]. They observed that the breaking force and deformation of the grass carp surimi gels increased significantly (*p* < 0.05) with the KGM concentration. Xiong *et al.* [12] also compared the breaking force and deformation of the gel sample between the control (without KGM) and high-level (2% KGM) samples: they found breaking force and deformation of 297.8 g and 6.8 mm in the control sample, while those of the gel sample with the highest level of KGM (2%) were 335.9 g and 8.13 mm, respectively. Xiong *et al.* [12] concluded that the textural properties of the surimigels could be affected by KGM, increasing the gel-forming ability and improving both surimi gel strength and elasticity. Zhou *et al.* [60] also noted that KGM could improve the gel properties of *Aristichthy nobilis* surimi. Therefore, KGM could be a potential enhancer of the gel properties in surimi processing. However, adding more than 2% KGM is not recommended because, at this level, the surimi gels could easily become too hard due to the strong hygroscopicity of KGM. Moreover, higher levels of KGM significantly reduced the whiteness of the surimi gels. The authors suggested that for a better surimi texture, the optimum level of KGM was 1%. 

## 5. Surimi-Hydroxypropyl Methylcellulose (HPMC) Mixtures

**Figure 3:**
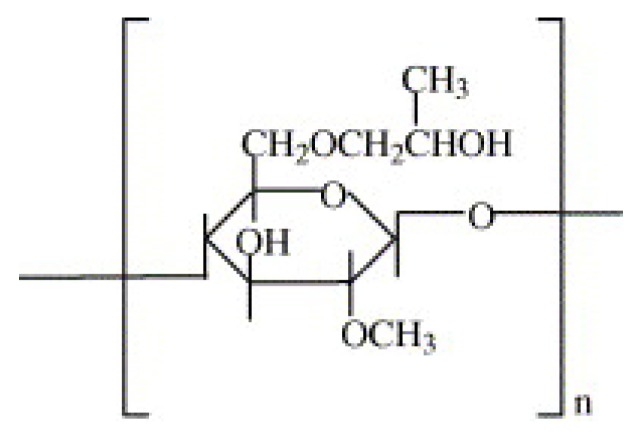
Structure of hydroxypropyl methylcellulose (adapted from Wang *et al*. [61]).

The structure of HPMC is presented in Figure 3. HPMC is soluble in cold water, but insoluble in hot water. HPMC is present in a variety of commercial products, such as ophthalmic lubricants and in excipient and controlled-delivery components in oral medications due to its semisynthetic, inert, viscoelastic, polymeric behavior. Moreover, HPMC is an emulsifier, thickening, and suspending agent and an alternative to animal gelatin. 

According to the report of Chen [62], HPMC had been used as a gelation-aid material in the preparation of horse mackerel surimi. It was found that the addition lowered the breaking force and enhanced the gel strength of heated surimi gel caused by the substantial rise in deformation [62]. Chen *et al.* [63] noted that, in HPMC-added surimi, HPMC form (powder or sol) and addition timing definitely influenced the structural geometry of the combination gel matrix, resulting in variations in rigidity, thermal stability and gel strength. Chen [64] used a thermal scanning rheological monitor and a rotational viscometer to perform small and large-strain tests, respectively, to observe concentration effect of the HPMC on the thermal rheological properties during the annealing process. The aim of the study was to obtain a reference for future investigation of the rheological properties of HPMC-enhanced surimi. The author used HPMC at concentrations of 2%, 5%, 10%, 15% and 20%, and noticed that in the large-strain test, at low concentrations of HPMC (2% and 5%) the apparent viscosity decreased with heating and then increased with cooling [64]. On the other hand, at high concentrations of HPMC (10%, 15% and 20%) the apparent viscosity was reversed, in that it increased with heating and then decreased with cooling. At 2% concentration HPMC behaved like a dilatant fluid. Gels containing 5% and 10% concentration of HPMC demonstrated pseudoplastic fluid behaviour, while gels containing 15% and 20% concentration HPMC demonstrated Bingham-pseudoplastic fluid behaviour. In the next study, Chen and Huang [13] used the thermal scanning rheological monitor and rotational viscometer for the purpose of studying the rheological properties of HPMC-enhanced surimi at various water contents. The addition of HPMC stabilized the surimi gel structures and improved flow properties. They concluded that HPMC gave surimi products a soft gelatinous texture.

## 6. Conclusions

The biological origin of starch has an important influence on the physico-chemical and functional properties of starch systems in surimi and its products. The viscoelastic moduli and the gel strength increased with increasing the starch concentration in surimi. The addition of combinations of cryoprotectants improved the rheological properties of surimi. The breaking force and deformation of the surimi gels increased significantly with the increasing addition of konjac glucomannan from 0 to 2% during frozen storage at −18 °C, which could also affect the textural properties of the surimigels. These phenomena could also increase the gel-forming ability and improve the strength and elasticity. HPMC is a useful gelation aid material to improve the flow properties of surimi.
